# Novel Sophoridine Derivatives Bearing Phosphoramide Mustard Moiety Exhibit Potent Antitumor Activities In Vitro and In Vivo

**DOI:** 10.3390/molecules23081960

**Published:** 2018-08-06

**Authors:** Dongdong Li, Linlin Dai, Xiumei Zhao, Shuang Zhi, Hongsheng Shen, Zibo Yang

**Affiliations:** Tianjin Institute of Medical and Pharmaceutical Sciences, Tianjin 300020, China; abcdll@whu.edu.cn (L.D.); zxmmlg@163.com (X.Z.); zhishuang0110@126.com (S.Z.); shenhongsheng830@163.com (H.S.); thesun999@163.com (Z.Y.)

**Keywords:** mustard functionalized sophoridine derivatives, antitumor activity, apoptosis

## Abstract

Novel mustard functionalized sophoridine derivatives were synthesized and evaluated for their cytotoxicity against of a panel of various cancer cell lines. They were shown to be more sensitive to S180 and H22 tumor cells with IC_50_ values ranging from 1.01–3.65 μM, and distinctly were more cytotoxic to cancer cells than normal cell L929. In addition, compounds **7a**, **7c**, and **7e** displayed moderate tumor suppression without apparent organ toxicity in vivo against mice bearing H22 liver tumors. Furthermore, they arrested tumor cells in the G1 phase and induced cellular apoptosis. Their potential binding modes with DNA-Top I complex have also been investigated.

## 1. Introduction

Natural phytochemicals have proven to be an extremely helpful tool to aid the discovery of potent antitumor agents for targets matching or tissue-selective therapy due to their privileged structures with inherent drug-likeness [1,2]. Sophoridine is a tetracyclic quinolizidine alkaloid extracted from the traditional medicine herb Sophora alopecuroides L. It was approved by China Food and Drug Administration (CFDA), in 2005, as an anticancer agent against malignant trophoblastic tumors [3]. Indeed, Sophoridine has been widely used as adjuvant with other anticancer pharmaceuticals including vinorelbine, cisplatin, and taxol to improve the therapeutic effect against gastric cancer, liver cancer, and esophageal cancer in combination therapy over time [4,5,6,7,8,9,10]. Sophoridine is also a main chemical ingredient of the Chinese traditional medicine *Fufang Kushen injection*, furthermore, it owns many of drugable advantages, such as high solubility, good safety profiles and a special chemical scaffold, indicating that it is an ideal lead compound for further investigation.

The mechanism of sophoridine is verified to inhibit the DNA topoiosmerase I activity, which induced cell cycle arrest in the G0/G1 phase and is accompanied by apoptotic cell death [11,12,13]. In addition to confirmed safety profiles, sophoridine exerts many favorable features such as potential of T cell improvement, good solubility, and no myelotoxicity [14,15,16]. However, the relatively moderate potency has generally limited its broader utility in clinic. Hence, considerable attentions have been devoted to discover sophoridine derivatives with optimized biochemical and/or pharmacological properties for new antineoplastic drug development. Concerning the unique scaffold of sophoridine, Xin Li et al. [17] have opened the sophoridine ring D and evaluated the resultant tricyclic sophoridinic acid. The result is that the sophoridine ring D might not be required for activity. The 12-nitrogen atom and 4′-carboxyl group of tricyclic sophoridinic acid provided modifiable sites for further diversity-oriented optimizations.

Cyclophosphamide is one of the most successful antitumor drugs for the management of malignancies of various origins from soft tissue tumor to lymphoma [18]. Because of its advantage of broad spectrum of antitumor and activity against both resting and multiplying cells, it still remains the drug of choice in routine clinical practices at this time [19]. Under physiological conditions, cyclophosphamide is metabolized to active phosphoramide mustard A, which is the ultimate cytotoxic species that cross-links interstrand DNA [20]. 

**Scheme 1:**
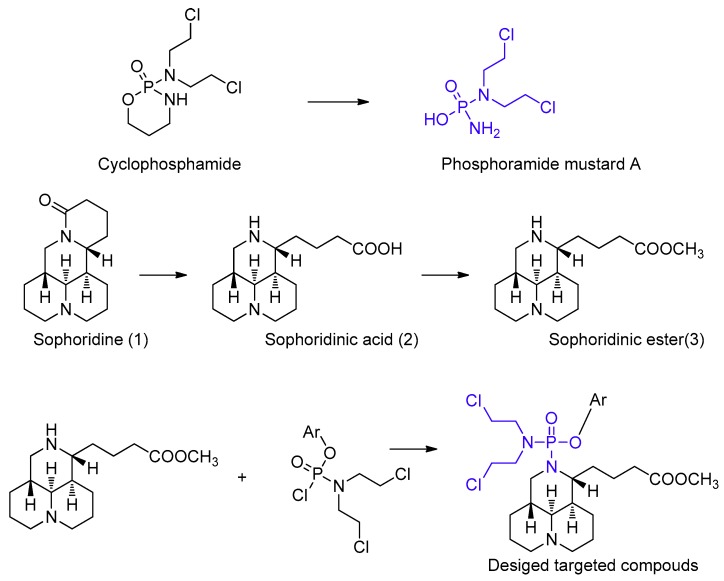
The formula structures of designed targeted compounds.

Based on the above suggestion, as depicted in Scheme 1, we have introduced phosphoramide mustard group to the structure of sophoridine, and designed and synthesized a series of novel sophoridine derivatives bearing phosphoramide mustard moiety. 

## 2. Results

### 2.1. Chemistry 

**Scheme 2:**
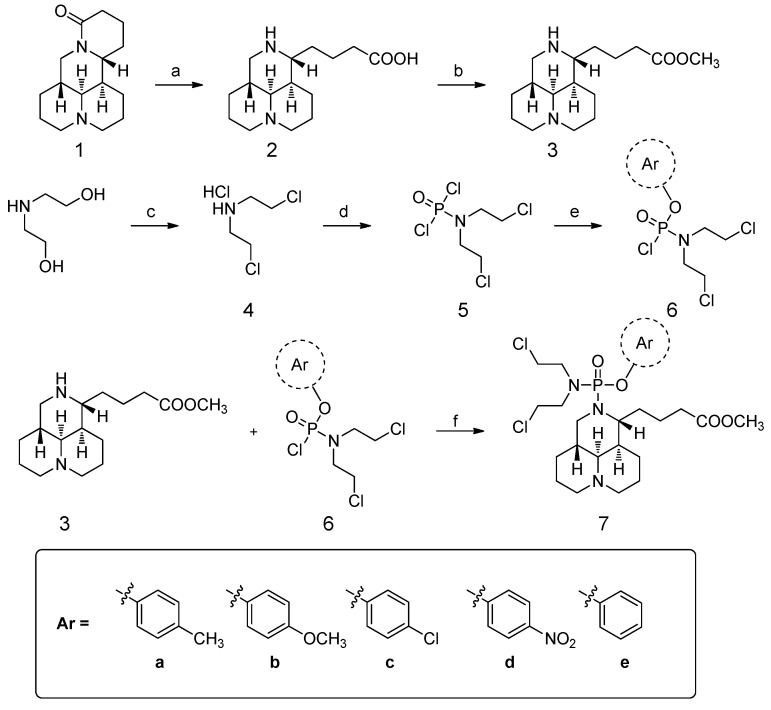
Synthesis of **7a**–**7e**. Reagents and conditions: (a) 2 N NaOH/H_2_O, reflux; 12N HCl; (b) anhydrous CH_3_OH, SOCl_2_/CH_3_OH; (c) SOCl_2_, CHCl_3_, ice bath then reflux; (d) excess POCl_3_, 120 °C; (e) substituted phenol, Et_3_N, anhydrous THF, ice bath; and (f) Et_3_N, anhydrous CH_3_CN, r.t.

Recently, we have designed and synthesized series of novel phosphoramide mustard sophoridine derivatives **7a**–**7e**, the synthetic approach consisted first of preparation of sophoridinic ester **2** and subsequent synthesis of the aryloxy-phosphoramidate mustard **6a**–**6e** in parallel. As depicted in Scheme 2, **1** was hydrolyzed in aqueous NaOH, and then acidified with 12 N HCl solution gave **2** with good yield. Intermediate **3** was afforded via esterification of **2** in thionyl chloride/methanol solution. **5** was prepared according to the previously reported protocols [21] as a crystalline solid from diethanolamine. Then we used the appropriate various phenols to react with **5** to obtain aryloxy-phosphoramidate mustards **6a**–**6e**. Finally, **6a**–**6e** was coupled with **3** in the presence of triethylamine, giving the target compounds **7a**–**7e** with satisfied yield. 

### 2.2. In Vitro Activity

**7a**–**7e** were evaluated for their antiproliferative activity against S180, H22, K562, MCF-7, SMMC-7721, and LoVo cells along with non-cancerous cells L929 by means of standard 3-(4,5-dimethyl-2-thiazolyl)-2,5-diphenyl-2-H-tetrazolium bromide (MTT) assay. Doxorubicin (Dox) was used as positive control, as illustrated in Table 1, higher activity was observed in some lineages evaluated. All the newly synthesized sophoridine derivatives were selectively efficacious against S180 and H22 cells with IC_50_ values in the range 1.01–3.65 µM regardless of the type of the substitutions on aryl. In S180 cells, compound **7c** (IC_50_, 2.02 μM), **7d** (IC_50_, 2.01 μM) and **7a** (IC_50_, 2.89 μM) were considered to be highly active. Another lineage sensitive to the compounds was H22 with three promising compounds **7c** (IC_50_, 1.01 μM), **7d** (IC_50_, 1.80 μM), and **7e** (IC_50_, 1.85 μM). The targeted compounds show more potency than the positive control Dox (IC_50_, 29.20 μM for S180, 36.51 μM for H22). Nonselective cytotoxicity is the main effect which hampers the use of optimal doses in most conventional chemotherapy [22]. Strikingly, the data suggest that derivatives **7a**–**7e** distinctly are more cytotoxic to cancer cells than the tested normal cell L929 according to the selectivity index (SI). 

Li et al. have reported a series of 12-N-substituted sophoridinic acid derivatives, structure activity relationships (SAR) analysis indicated that the substituents introduced on the 12-nitrogen atom might significantly enhance the anticancer activity of this kind of compounds, especially, compound **6b** (30 µg/mL), possessing a bromoacetly group, afforded the potential antiproliferative activities with inhibition rate of 88%, 68%, 40%, and 73% against HepG2, HCT1116, A549, and MCF-7 cells, respectively, much stronger than that of sophoridine. In this work, sophoridine derivatives bearing phosphoramide mustard moiety **7a**–**7e** have also exhibited much stronger than the lead compound with IC_50_ values ranging from 1.01–3.65 μM against S180 and H22 tumor cells.

We also tested the kinetics of growth inhibition of H22 and S180 cell lines for **7c** and **7d** at the dosages of 1–20 µM. Seen in Figure S1, they all showed concentration-dependent and time-dependent on inhibitory effect. 

### 2.3. In Vivo Activity

**Figure 1:**
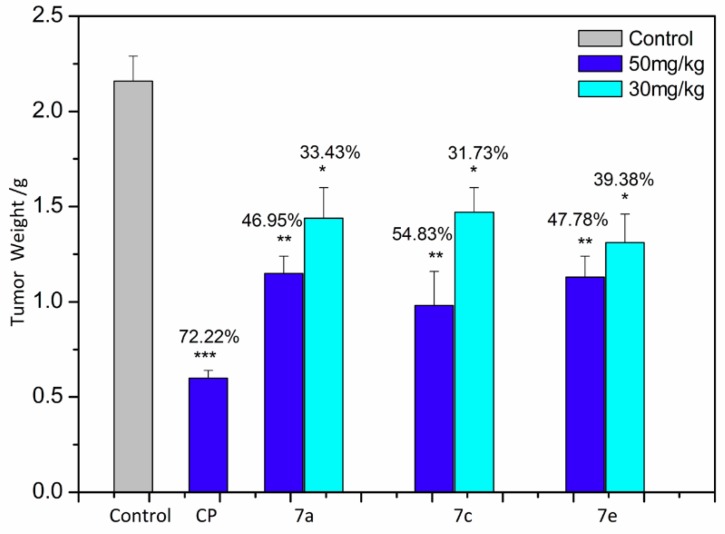
The effect of different dosage of **7a**, **7c**, and **7e** on the in vivo tumor growth of H22 mice. Normal saline (NS) and cyclophosphamide (CP) were utilized as a negative control and positive control, respectively. * *p* < 0.05, * *p* < 0.01, *** *p* < 0.001 compared with untreated control group.

Based on the above suggestion, **7a**, **7c**, and **7e** were selected for evaluating the antitumor activity in H22 tumor bearing mice models. Twenty-four hours after implantation, the mice were randomly divided into eight experimental groups. Normal saline (NS) and cyclophosphamide (CP) were utilized as a negative control and positive control, respectively. For the tested compounds, the cytotoxicity against H22 indicated **7c** (IC_50_: 1.01 μM) > **7e** (IC_50_: 1.85 μM) > **7a** (IC_50_: 2.17 μM), and the tumor inhibition rate showed **7c** (54.83%) > **7e** (47.78%) > **7a** (46.95%) at the dose of 50 mg/kg. This suggested that the in vivo antitumor potency correlated well with their in vitro antiproliferative activities. Compared with CP groups, though their activities were weaker than that of positive control CP (72.22%), **7a**, **7c**, and **7e** did not significantly cause body weight change comparing with the NS group even at the dose of 50 mg/kg. Compared with NS group, **7a**, **7c** and **7e** treated groups showed similar effect at the indicated dosage, there was no obvious different between **7c**, **7a**, and **7e**, this indicated that **7a** and **7c** containing electron-donating groups did not enhance binding affinity with corresponding receptor in vivo compared with **7e**. For **7a**, **7c**, and **7e** treated groups, the tumor inhibitory effects of high dose were superior to that of low dose in all treated groups, and the reduction of tumor growth depends on dosage. Seen in Figure 1.

**Figure 2:**
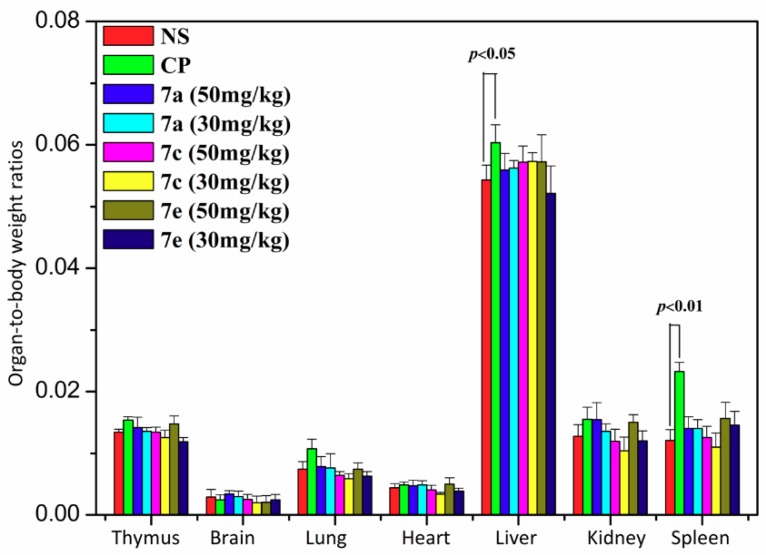
Effects of different dosage of **7a**, **7c**, and **7e** on organ index in H22 bearing mice. Normal saline (NS) and cyclophosphamide (CP) were utilized as a negative control and positive control, respectively. *p* < 0.05, *p* < 0.01 compared with untreated control group.

To evaluate the toxicity of **7a**, **7c**, and **7e**, necropsy of the dead mice was carried out. Organ-to-body weights ratios were compared between treated (50 and 30 mg/kg dose) and vehicle groups of animals, the data were listed in Figure 2. The thymus, brain, lung, heart, liver, kidney, and spleen indexes of tested compounds were comparable to that of NS, and did not show any significant difference. Compared with NS, CP was found to significantly increase the liver and spleen indexes, suggesting that CP could cause change in liver (*p* < 0.05) and spleen (*p* < 0.01) function of H22 mice. The results indicated that **7a**, **7c**, and **7e** induced low toxicity to the mice in vivo.

### 2.4. Apoptosis Analysis

**Figure 3:**
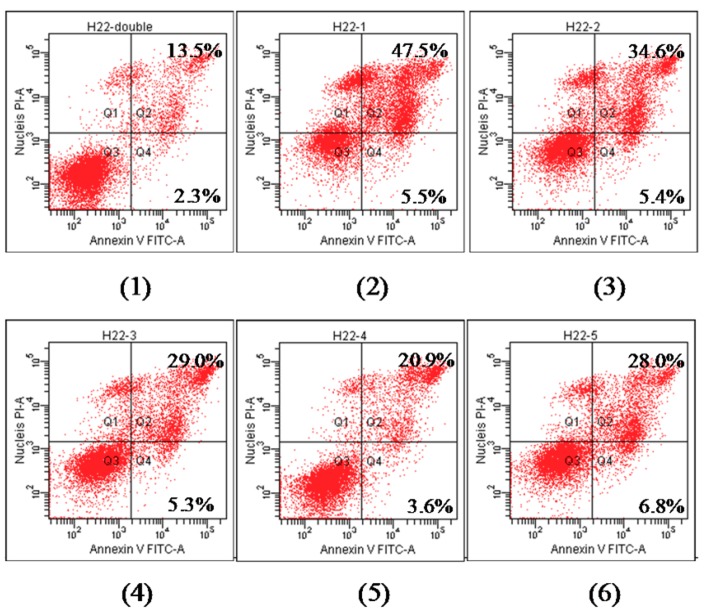
Effects of **7a**–**7e** on cell apoptosis of H22 for 48 h: (**1**) control; (**2**) **7a**, 3 μM; (**3**) **7b**, 3 μM; (**4**) **7c**, 3 μM; (**5**) **7d**, 3 μM; and (**6**) **7e**, 3 μM.

We use an annexin V-FITC/PI apoptosis detection kit to evaluate the efficacy of **7a**–**7e** on H22 tumor cell line. The result is shown in Figure 3. Compared with the negative control, **7a**–**7e** increased the apoptosis at the concentration of 3 μM for 48 h, the order of the ability to induce apoptosis is **7a** > **7b** > **7e** > **7c** > **7d**. The results demonstrated that the antitumor activity of targeted compounds was due to the induction of apoptosis in H22 tumor cell line.

### 2.5. Cell Cycle Analysis

**Figure 4:**
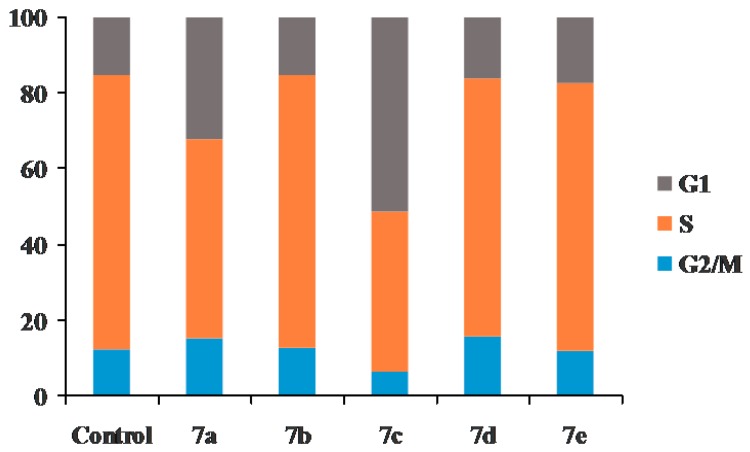
Cell cycle analysis by flow cytometry of H22 cells treated with **7a**–**7e** for 48 h: (1) control; (2) **7a**, 3 μM; (3) **7b**, 3 μM; (4) **7c**, 3 μM; (5) **7d**, 3 μM; and (6) **7e**, 3 μM.

To determine whether the suppression of cell growth by the compounds was caused by a cell-cycle effect, we used a cell cycle analysis kit to detect the DNA content of cell nuclei. As shown in Figure 4, when H22 cells were treated with **7a**–**7e** at the concentration of 3 μM for 48 h, the percentage of G1-phase cells increased from 15.32% to 32.02% associated with a percentage decrease of S-phase cells (72.42% to 52.83%) for **7a**, the percentage of G1-phase cells increased from 15.32% to 15.44% associated with a percentage decrease of S-phase cells (72.42% to 71.86%) for **7b**, the percentage of G1-phase cells increased from 15.32% to 51.22% associated with a percentage decrease of S-phase cells (72.42% to 42.48%) for **7c**, the percentage of G1-phase cells increased from 15.32% to 16.10% associated with a percentage decrease of S-phase cells (72.42% to 68.24%) for **7d**, and a percentage increase of G1-phase cells (15.32% to 17.21%) associated with a percentage decrease of S-phase cells (72.42% to 71.04%) for **7e**. Cell cycle analysis showed that the compounds all induced G1 cell cycle arrest in H22 cells, especially for **7a** and **7c**.

### 2.6. Molecule Docking 

**Figure 5:**
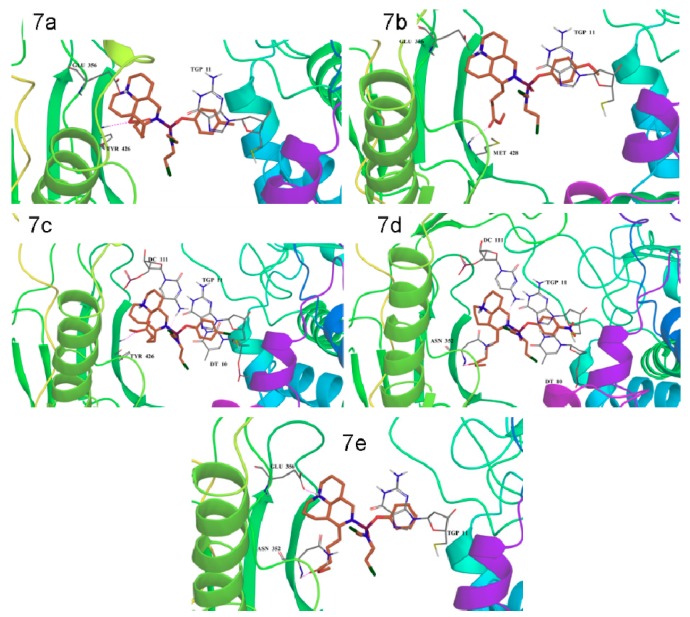
Schematic representation of the proposed binding mode for **7a**–**7e** with Top I-DNA (Protein Data Bank (PDB) code: 1T8I).

Previously, Top I was found to be the antitumor target of sophoridine derivatives [23,24]. Typical Top I inhibitor camptothecin acts by stabilizing a covalent Top I-DNA complex called the cleavable complex. Molecular docking studies were performed to find out the possible binding mode of the compounds with DNA-Top I complex (Protein Data Bank (PDB) ID: 1T8I). **7a**–**7e** was chosen for the docking analysis. As seen in Figure 5, alkyl ester chain and parts of tricycle scaffold of the compound is buried in the hydrophobic pocket by ILE427, TYR426, LEU429, ALA351, PRO431, and MET428 residues. Base-stacking interactions (π–π) are formed between the substituted aryl and the surrounding DNA base pair TGP11. The carbonyl oxygen atom of **7a**–**7e** formed a hydrogen bond with TYR426, MET428, TYR426, ASN352, and ASN352, respectively. Moreover, the protonated-cationic nitrogen at the bottom of the tricycle scaffold for **7a**, **7b**, and **7e** is involved in a hydrogen bond with the GLU365 or LEU356 residue. The halogen bond for **7c** and **7d** is also observed. The docking statistics was seen in Table S2. Camptothecin is the positive control, the docking results show that the stability between **7a**–**7e** and DNA-Top I is more potent than that of CPT, indicating that the compounds have interaction with DNA-Top I [25], the result suggested that the chemical modification retains the mode of action of its parent.

## 3. Materials and Methods 

### 3.1. Instrumentation

All the compounds were characterized with ^1^H-NMR, ^13^C-NMR and ESI-MS. Nuclear magnetic resonance data were recorded on spectrometer (Bruker Avance 400, Billerica, MA, USA) with CDCl_3_ as solvent, tetramethylsilane (TMS) as an internal standard. Mass spectra were obtained from an LCMS-8040 (SHIMADZU, Kyōto, Japan). The optical density was measured at the 570 nm wavelength on a multimode reader (Tecan Infinite M200, Zürich, Switzerland). The fluorescence intensity was measured with a Flow Cytometer (BD FACSCanto II, East Rutherford, NJ, USA).

### 3.2. Materials

Sophoridine (98%) was obtained from Shanghai PureOne Biotechnology Co., Ltd. (Shanghai, China). All the other chemicals reagents used in the experiments were of analytical grade and obtained from Tianjin Chemical Reagent Co., Ltd. (Tianjin, China). Ethyl acetate, methanol, and petroleum ether were distilled before use. Chloroform, acetonitrile and triethylamine were distilled from CaH_2_. Tetrahydrofuran was purified by distillation under Ar atmosphere from Na/benzophenone immediately prior to use.

### 3.3. Synthesis of Compounds

#### 3.3.1. Synthesis of **2**

Sophoridine **1** (0.03 mol, 7.44 g) was dissolved in 2 N sodium hydroxide solution and heated to reflux for several days, after cooling down, the pH of the mixture was adjusted to 5–6 with 20% H_2_SO_4_ and then filtered. The solution was evaporated under vacuum and the resultant solid was crystallized in methanol, the yellow solid was obtained and dried under vacuum. Yield (85%), m.p. 154~155 °C. C_15_H_26_N_2_O_2_ (266.4): MS(ESI) *m*/*z*: [M + H]^+^ = 267.2, [M − H]^−^ = 265.2.

#### 3.3.2. Synthesis of **3**

To a stirred anhydrous methanol solution of **2** (0.02 mol, 5.33 g), the anhydrous methanol solution of SOCl_2_ was added dropwise under ice bath. The mixture was stirred for 4 h and evaporated under vacuum and light yellow solid was obtained, and then recrystallized in methanol-acetone, white solid was obtained and dried under vacuum. Yield (63%). ^1^H-NMR (400 MHz, CDCl_3_) δ: 11.55 (s, 1H), 9.43 (d, *J* = 38.0 Hz, 2H), 3.88 (s, 1H), 3.73 (d, *J* = 28.2 Hz, 4H), 3.53–3.34 (m, 3H), 3.21 (d, *J* = 46.1 Hz, 2H), 2.82 (d, *J* = 60.2 Hz, 3H), 2.35 (d, *J* = 65.8 Hz, 4H), 1.97 (d, *J* = 45.2 Hz, 9H), 1.70 (d, *J* = 11.0 Hz, 1H), 1.49 (s, 1H); ^13^C-NMR δ: 173.11, 57.98, 56.84, 54.93, 52.82, 51.76, 45.41, 42.88, 33.82, 33.13, 27.70, 25.60, 22.35, 22.16, 21.56, 17.92; C_16_H_28_N_2_O_2_ (280.4) MS(ESI) *m*/*z*: [M + H]^+^ = 281.2.

#### 3.3.3. Synthesis of **4**

To a stirred chloroform solution of diethanol amine (0.20 mol, 21.01 g), the chloroform solution of SOCl_2_ was added dropwise under ice-bath. The mixture was stirred for 6 h and evaporated under vacuum and white solid was obtained, and then recrystallized in absolute alcohol, white needle solid was obtained and dried under vacuum. Yield (50%). m.p. 215~216 °C. C_4_H_10_NCl_3_ (178.5), MS(ESI) *m*/*z*: [M − HCl + H]^+^ = 142.0.

#### 3.3.4. Synthesis of **5**

Complex **4** (0.056 mol, 10.00 g) was resolved in 26 mL POCl_3_, and heated under reflux until complete solution resulted. The excess POCl_3_ was removed by distillation and the residue distilled at reduced pressure. The oil was crystallized as a solid mass on cooling, dried under vacuum. Yield (74%). m.p. 53~54 °C. ^1^H-NMR (400 MHz, CDCl_3_) δ 3.74 (dd, *J* = 9.9, 4.8 Hz, 4H), 3.68 (dd, *J* = 14.4, 8.6 Hz, 4H).

#### 3.3.5. Synthesis of **6a**–**6e**

To a stirred solution of 4-methyl phenol (10 mmol, 1.08 g) and **5** (12 mmol, 3.10 g) in anhydrous THF (30 mL) at 70 °C oil bath, Et_3_N was added dropwise. The reaction solution was refluxed till TLC analysis showed completion of the reaction and filtered, the filtrate was evaporated under vacuum and purified by column chromatography on the silica gel with petroleum ether-dichloromethane/triethylamine as eluents to give compound **6a** as yellow oil in 61% yield. 

Compound **6b**–**6e** was prepared in the same manner as described above.

**6a**: Yield: 61%; yellow oil; ^1^H-NMR (400 MHz, CDCl_3_) δ: 7.19 (d, *J* = 8.6 Hz, 2H), 7.14 (d, *J* = 8.6 Hz, 2H), 3.72 (d, *J* = 5.3 Hz, 8H), 2.35 (s, 3H); ^13^C-NMR δ: 147.20, 136.10, 136.02, 130.50, 120.15, 120.10, 49.79, 49.31, 41.48, 40.81, 20.83; ESI-MS: C_11_H_15_Cl_3_NO_2_P (330.6), [M + Na]^+^ = 354.0.

**6b**: Yield: 68%; yellow oil; ^1^H-NMR (400 MHz, CDCl_3_) δ: 7.19 (dd, *J* = 9.1, 1.8 Hz, 2H), 6.90 (d, *J* = 8.8 Hz, 2H), 3.82 (s, 3H), 3.78–3.71 (m, 4H), 3.69–3.57 (m, 4H); ^13^C-NMR δ: 157.55, 143.07, 121.40, 121.35, 114.90, 114.89, 55.64, 49.87, 49.83, 41.46, 41.44; ESI-MS: C_11_H_15_Cl_3_NO_3_P (346.6), [M + Na]^+^ = 370.1.

**6c**: Yield: 62%; yellow oil; ^1^H-NMR (400 MHz, CDCl_3_) δ: 7.38 (d, *J* = 8.5 Hz, 2H), 7.23 (d, *J* = 7.3 Hz, 2H), 3.74 (dd, *J* = 9.7, 2.6 Hz, 4H), 3.65 (dd, *J* = 14.4, 7.1 Hz, 4H); ^13^C-NMR δ: 148.04, 131.79, 131.76, 130.11, 121.87, 121.81, 49.75, 49.71, 41.38, 41.36. ESI-MS: C_10_H_12_Cl_4_NO_2_P (351.0), [M + Na]^+^ = 374.0.

**6d**: Yield: 53%; yellow oil; ^1^H-NMR (400 MHz, CDCl_3_) δ: 8.31 (d, *J* = 9.1 Hz, 2H), 7.46 (d, *J* = 8.9 Hz, 2H), 3.78–3.60 (m, 8H); ^13^C-NMR δ: 154.89, 145.11, 142.40, 126.17, 125.89, 120.71, 120.65, 49.17, 49.13, 41.57. ESI-MS: C_10_H_12_Cl_3_N_2_O_4_P (361.5), [M + Na]^+^ = 385.2.

**6e**: Yield: 65%; yellow oil; ^1^H-NMR (400 MHz, CDCl_3_) δ: 7.42 (t, *J* = 7.5 Hz, 2H), 7.29 (d, *J* = 1.6 Hz, 2H), 3.75 (dd, *J* = 7.0, 2.1 Hz, 4H), 3.66 (dd, *J* = 19.1, 11.3 Hz, 4H); ^13^C-NMR δ: 150.1, 130.08, 130.10, 126.25, 120.47, 120.42, 49.88, 49.84, 41.46, 41.44. ESI-MS: C_10_H_13_Cl_3_NO_2_P (316.5), [M + Na]^+^ = 340.1.

#### 3.3.6. Synthesis of **7a**–**7e**

To a stirred solution of **6a** (0.30 mmol, 0.10 g) and **3** (0.56 mmol, 0.20 g) in anhydrous CH_3_CN (30 mL) at 80 °C oil bath, Et_3_N (3.0 mmol, 0.3 g) in 10 mL anhydrous CH_3_CN was added dropwise. The reaction solution was refluxed till TLC analysis showed completion of the reaction and filtered, the filtrate was evaporated under vacuum and purified by column chromatography on the silica gel to obtain colorless oil **7a**. 

**7b**–**7e** was prepared in the same manner as described above.

**7a**: Yield: 39%; colorless oil; ^1^H-NMR (400 MHz, CDCl_3_) δ: 7.15 (d, *J* = 3.4 Hz, 2H), 7.11 (d, *J* = 6.2 Hz, 2H), 3.68 (d, *J* = 2.4 Hz, 2H), 3.57 (d, *J* = 6.5 Hz, 2H), 3.43 (d, *J* = 7.0 Hz, 2H), 3.05–2.80 (m, 4H), 2.62–2.44 (m, 4H), 2.44–2.09 (m, 8H), 2.08–1.98 (m, 2H), 1.81 (dd, *J* = 8.9, 4.1 Hz, 2H), 1.68 (dt, *J* = 11.4, 6.3 Hz, 6H), 1.49 (t, *J* = 7.3 Hz, 2H), 1.45–1.27 (m, 4H); ^13^C-NMR δ: 173.46, 130.17, 130.10, 120.03, 119.98, 119.81, 119.76, 58.98, 58.54, 54.45, 51.56, 50.30, 49.97, 45.41, 44.32, 42.44, 38.58, 33.86, 33.68, 30.74, 29.16, 26.99, 25.73, 24.74, 22.29, 20.80, 18.97; ESI-MS: C_27_H_42_Cl_2_N_3_O_4_P (574.5), [M + H]^+^ = 575.2.

**7b**: Yield: 41%; colorless oil; ^1^H-NMR (400 MHz, CDCl_3_) δ: 7.15 (dd, *J* = 25.4, 8.7 Hz, 2H), 6.84 (dd, *J* = 9.1, 3.4 Hz, 2H), 3.78 (s, 3H), 3.65 (s, 3H), 3.54 (dd, *J* = 8.8, 2.9 Hz, 2H), 3.40 (dd, *J* = 6.9, 4.8 Hz, 2H), 3.20–3.12 (m, 2H), 3.04–2.86 (m, 3H), 2.85–2.73 (m, 2H), 2.62–2.36 (m, 3H), 2.37–2.06 (m, 3H), 1.91–1.70 (m, 5H), 1.70–1.57 (m, 6H), 1.56–1.39 (m, 2H), 1.28 (dd, *J* = 21.8, 13.5 Hz, 2H). ^13^C-NMR δ: 173.62, 156.46, 144.88, 121.13, 120.93, 114.62, 114.59, 58.88, 58.54, 58.40, 55.67, 54.55, 51.66, 50.35, 49.95, 45.31, 42.29, 38.71, 37.88, 33.82, 30.85, 29.31, 26.87, 25.98, 24.92, 22.54, 19.02. ESI-MS: C_27_H_42_Cl_2_N_3_O_5_P (590.5), [M + H]^+^ = 592.3.

**7c**: Yield: 42%; colorless oil; ^1^H-NMR (400 MHz, CDCl_3_) δ: 7.26 (dd, *J* = 8.9, 4.4 Hz, 2H), 7.21 (d, *J* = 8.9 Hz, 1H), 7.15 (d, *J* = 8.5 Hz, 1H), 3.63 (s, 3H), 3.47 (dddd, *J* = 26.8, 20.3, 14.8, 7.3 Hz, 9H), 3.14 (td, *J* = 9.5, 4.8 Hz, 1H), 2.96–2.75 (m, 4H), 2.60–2.21 (m, 4H), 1.99–1.21 (m, 13H), 1.02 (t, *J* = 7.2 Hz, 1H); ^13^C-NMR δ: 173.60, 149.90, 129.65, 121.60, 121.38, 77.44, 77.12, 76.80, 58.80, 58.65, 58.46, 54.50, 51.53, 51.51, 50.26, 50.22, 49.87, 49.83, 46.17, 45.35, 45.28, 44.95, 44.40, 44.35, 42.36, 42.21, 38.66, 37.88, 37.83, 33.71, 33.57, 30.80, 30.73, 30.68, 29.30, 29.27, 26.87, 25.89, 24.98, 24.91, 22.48, 22.32, 18.97. ESI-MS: C_26_H_39_Cl_3_N_3_O_4_P (595.0), [M + H]^+^ = 596.2.

**7d**: Yield: 31%; light yellow oil; ^1^H-NMR (400 MHz, CDCl_3_) δ: 8.17 (d, *J* = 6.9 Hz, 2H), 7.38 (dd, *J* = 17.7, 8.9 Hz, 2H), 3.59 (d, *J* = 4.0 Hz, 3H), 3.48–3.35 (m, 5H), 3.17 (d, *J* = 9.9 Hz, 1H), 2.87 (dt, *J* = 32.4, 14.3 Hz, 4H), 2.70–2.05 (m, 6H), 1.97–1.85 (m, 1H), 1.79–1.15 (m, 15H); ^13^C-NMR δ: 172.56, 155.15, 143.28, 124.68, 124.66, 119.63, 119.44, 57.82, 57.45, 53.42, 50.61, 50.57, 49.13, 49.09, 48.75, 48.70, 44.34, 41.36, 41.18, 37.51, 32.48, 28.09, 26.07, 24.65, 23.83, 21.31. ESI-MS: C_26_H_39_Cl_2_N_4_O_6_P (605.5), [M + H]^+^ = 606.3.

**7e**: Yield: 39%; colorless oil; ^1^H-NMR (400 MHz, CDCl_3_) δ: 7.37 (d, *J* = 6.7 Hz, 2H), 7.32 (d, *J* = 5.8 Hz, 1H), 7.26 (d, *J* = 7.7 Hz, 1H), 7.20 (t, *J* = 7.0 Hz, 1H), 3.70 (s, 3H), 3.60 (qd, *J* = 17.8, 10.8 Hz, 5H), 3.47 (d, *J* = 6.3 Hz, 4H), 3.23 (t, *J* = 11.6 Hz, 1H), 2.90 (ddd, *J* = 26.2, 24.5, 12.3 Hz, 4H), 2.66–2.21 (m, 5H), 2.14–0.83 (m, 13H). ^13^C-NMR δ: 173.73, 150.79, 129.75, 129.65, 124.63, 120.37, 120.08, 58.94, 58.43, 58.43, 54.56, 51.59, 50.31, 49.96, 44.45, 42.38, 38.69, 37.89, 33.69, 30.88, 29.29, 26.98, 25.93, 24.96, 22.57, 19.03. ESI-MS: C_26_H_40_Cl_2_N_3_O_4_P (560.5), [M + Na]^+^ = 582.2. 

### 3.4. Anti-Proliferation Assay

The ability of anti-proliferation of compounds was evaluated by MTT assay. Targeted tumor cell lines were grown in an RPMI-1640 medium supplemented with 10% fetal bovine serum. The cells were incubated at 37 °C under a 5% CO_2_ atmosphere for 24 h prior to the addition of the compounds.

All the targeted compounds were dissolved in DMSO as stock solution. After a 48 h exposure period, PBS containing MTT was added to each well for 4 h, the media with MTT were removed, and DMSO was added, and then measured by UV spectrometer at 570 nm using a multimode reader (Tecan infinite M200, Zürich, Switzerland), all experiments were performed in triplicate.

### 3.5. Measurement of Apoptosis by Annexin V Analysis

H22 Cells were seeded in a 6-well culture plate for 12 h at 37 °C and 5% CO_2_, and **7a**–**7e** were added to each well respectively at 3 μM and incubated for 48 h. The H22 cells were then harvested by trypainization and washed twice with cold PBS, centrifugated, resuspended in binding buffer which was then added to 5 μL of annexin V-FITC and incubated at room temperature for 15 min. After adding 10 μL of PI, the cells were incubated for another 15 min in dark. The stained cells were analyzed by a Flow Cytometer and the average percentage of Annexin V-positive cells was used as a measure of apoptosis.

### 3.6. Cell Cycle Analysis

H22 cells were treated with **7a**–**7e** at 3 μM at 37 °C for 48 h and then cells were harvested, centrifuged, resuspended and fixed in 70% EtOH overnight at 4 °C. The fixed cells were digested by RNase A (0.25 mg·mL^−1^) at 37 °C for 30 min, and stained with PI (50 μg·mL^−1^) in the dark at room temperature for 30 min. The distribution of the cell cycle was analyzed by Flow Cytometer.

### 3.7. Molecular Docking

The chemical structures of **7a**–**7e** were drawn with chemoffice, protonated using the protonate 3D protocol and subjected to an energy minimization with Ligand Preparation of Schrödinger using OPLS_2005 force field. Then, all of the conformations of the studied compounds were generated for further docking. The crystal structure of DNA-Top I in complex with camptothecin (PBD ID: 1T8I) was obtained from Protein Data Bank. Water molecules in the 1T8I.pdb file were deleted, bond orders were assigned, and hydrogen atoms were added to the protein. And then the protein was prepared using protein preparation and refinement tool. For the active site, a grid box centered at the ligand camptothecin (EHD) was used to accommodate a maximum ligand length of 15 Å. Docking all conformations of ligands flexibly with extra precision (XP) mode of Glide of default parameters, and writing out a maximum of two poses per ligand, and also enabling the post-docking minimization of the ligands. Glide score and glide energy are used as scoring functions. 

### 3.8. Antitumor Activity In Vivo

Tumor-bearing mice were prepared by inoculating a suspension of H22 cells (1.2 × 10^6^ cells per mouse) subcutaneously into the right armpit of KM mousse, five days inoculation, according to body weight, 40 mice were assigned to eight equal groups: i.v. Cyclophosphamide group (positive control, 50 mg/kg), **7a** (50 mg/kg), **7a** (30 mg/kg), **7c** (50 mg/kg), **7c** (30 mg/kg), **7e** (50 mg/kg), **7e** (30 mg/kg), and isotonic saline group (negative control). All treatments were administered for 6 days. One day after, mice were sacrificed, the body, neoplasm and organ weight were measured. The inhibition was calculated as follows: inhibition (%) = [1 − (mean tumor weight of tested mice)/(mean tumor weight of control mice] × 100.

All in vivo assays were performed in the Central Laboratory of the Tianjin Institute of Medical and Pharmaceutical Sciences, and approved by the Committee on Ethics of the Tianjin Institute of Medical and Pharmaceutical Sciences. The ethical approval code is IMPS-EAEP-Z-17JCQNJC13600.

## 4. Conclusions

In conclusion, a series of novel phosphoramide mustard functionalized sophoridine derivatives were evaluated for their cytotoxic potential against a panel of various cancer cell lines. All targeted compounds bearing this type of scaffold are more sensitive to S180 and H22 cells with IC_50_ values ranging from 1.01–3.65 μM. Substitution on the aryl affects the activity of the compounds, while this difference in activity is modest and within the same order of magnitude. All targeted derivatives distinctly are more cytotoxic to cancer cells than the tested non-cancerous cell L929. Compound **7a**, **7c**, and **7e** were selected for further antitumor therapeutic efficacy in vivo against mice bearing H22 liver cancer. Compared with vehicle group, the selected compounds showed moderate tumor suppression and are of low organ toxicity. Moreover, they could induce apoptosis and lead to G1 cell cycle arrest in H22 tumor cells, molecular docking studies revealed that these compounds have interaction with DNA-Top I, so they could be considered as the potent targeted antitumor agents to further develop.

## Figures and Tables

**Table 1:** Antitumor effect of **7a**–**7e** in vitro (48 h).

Compound	Cell Lines (IC50, μM)								
	S180	H22	K562	MCF-7	SMMC-7721	LoVo	L929	SI *	SI **
**5**	12.13 ± 0.55	11.70 ± 1.32	48.76 ± 2.01	111.37 ± 3.95	48.76 ± 2.74	83.98 ± 2.36	52.84 ± 2.80	4.36	4.52
**7a**	2.89 ± 0.23	2.17 ± 0.13	27.07 ± 2.51	61.20 ± 2.21	60.80 ± 2.34	62.47 ± 2.32	180.66 ± 4.56	62.51	83.10
**7b**	3.50 ± 0.35	2.65 ± 0.25	54.50 ± 2.32	38.86 ± 2.56	65.16 ± 2.67	46.17 ± 2.32	104.30 ± 3.20	29.80	39.36
**7c**	2.02 ± 0.17	1.01 ± 0.08	18.07 ± 1.34	31.92 ± 2.45	31.73 ± 1.21	33.43 ± 2.45	162.41 ± 4.12	80.40	160.80
**7d**	2.01 ± 0.14	1.80 ± 0.12	20.31 ± 2.05	34.62 ± 2.21	36.51 ± 2.45	32.25 ± 2.21	105.32 ± 3.10	52.40	58.51
**7e**	3.65 ± 0.25	1.85 ± 0.16	32.73 ± 2.56	86.57 ± 2.67	77.89 ± 2.78	69.95 ± 2.78	138.54 ± 3.35	37.96	74.89
Dox	29.20 ± 2.07	36.51 ± 2.56	17.23 ± 1.78	0.44 ± 0.08	1.22 ± 0.05	0.15 ± 0.02	0.23 ± 0.08	-	-
1	>100	>100	>100	>100	>100	>100	>100		

IC_50_ values were expressed as mean values. * Indicates a selectivity index (SI) of S180 against L929. ** Indicates a selectivity index (SI) of H22 against L929.

## Supplementary Materials

The following are available online. The synthesis and characterization of **2**, **3**, **4**, **5**, and **6a**–**6e**, time-dependent growth inhibition of **7c** and **7d** on H22 and S180 cell lines, cell cycle inhibition in H22 cell line by treating with **7a**–**7e**, and docking statistics of **7a**–**7e** against DNA-Top I.
