# Supplementary material for: Novel Sophoridine Derivatives Bearing Phosphoramide Mustard Moiety Exhibit Potent Antitumor Activities In Vitro and In Vivo
Source: Molecules. 2018 Aug 6;23(8):1960. doi: 10.3390/molecules23081960 (PMC6222802; doi:10.3390/molecules23081960)
Supplement: Supplementary file 1 [file molecules-23-01960-s001.pdf]

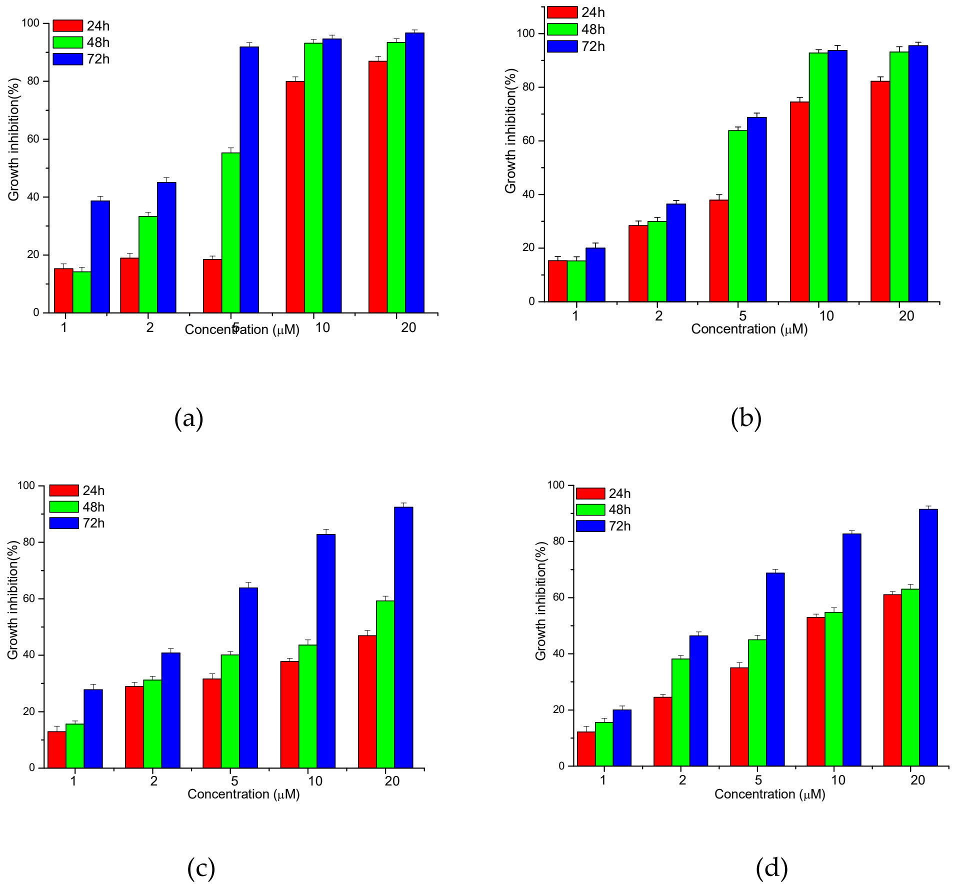

Figure S1. Time-dependent growth inhibition of **7c** and **7d** on H22 and S180 cell lines. Cells were cultured for 24–72h at concentrations of 1–20 μM. (a)**7c**, H22; (b)**7c**, S180; (c) **7d**, H22; (d) **7d**, S180.

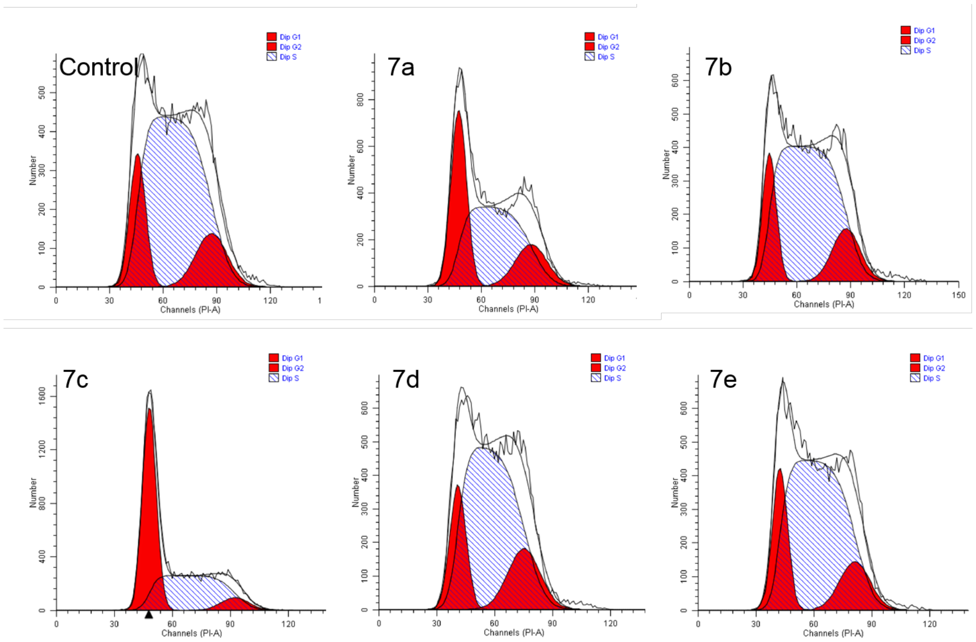

Figure S2. Cell cycle inhibition in H22 cell line by treating with compounds **7a**–**7e** for 48 h.

Table S1. Cell cycle analysis by flow cytometry of H22 cells treated with compounds **7a**–**7e** for.48 h.

**Sample**	**Distribution (%)**		
	**G1**	**S**	**G2/M**
**Control**	15.32	72.42	12.26
**7a**	32.02	52.83	15.15
**7b**	15.44	71.86	12.70
**7c**	51.22	42.48	6.30
**7d**	16.10	68.24	15.66
**7e**	17.21	71.04	11.75

Table S2. Docking statistics of synthesized compounds **7a**–**7e** against DNA-Top I.

**Compound**	**Glide Score**	**Glide Energy**	**H-bonding Ligand/ Receptor**	**π-π Interaction**
**CPT**	-8.704	-88.330	N of quinoline group/ H of ARG364; H of hydroxyl group / O of ASP533	CPT/TGP11 and DT10 of DNA
**7a**	-9.614	-98.615	O of carbonyl group / H of TYR426; H of ammonium group / O of GLU356	Aromatic group of compound/TGP11 of DNA
**7b**	-9.999	-108.058	O of carbonyl group / H of MET428; H of ammonium group / O of LEU356	Aromatic group of compound/TGP11 of DNA
**7c**	-8.793	-104.546	O of carbonyl group / H of TYR426; Cl of mustard nitrogen / H of DC111	Aromatic group of compound/TGP11 and DT10 of DNA
**7d**	-8.920	-110.507	O of carbonyl group / H of ASN352; Cl of mustard nitrogen / H of DC111	Aromatic group of compound/TGP11 and DT10 of DNA
**7e**	-9.565	-93.638	O of carbonyl group / H of ASN352; H of ammonium group / O of GLU356	Aromatic group of compound/TGP11 of DNA
